# Prevalence of myopia in 3–14-year-old Chinese children: a school-based cross-sectional study in Chengdu

**DOI:** 10.1186/s12886-021-02071-6

**Published:** 2021-09-01

**Authors:** Jianglan Wang, Jinnan Liu, Wei Ma, Qi Zhang, Rong Li, Xiao He, Longqian Liu

**Affiliations:** 1 Department of Optometry and Vision Science, West China School of Medicine, Sichuan University, 37 Guoxue Xiang, Sichuan Province Chengdu, China; 2 EyeSee Medical Science & Technology Chengdu Co, Ltd. 49 Dongfu street, Jinjiang District, Chengdu, Sichuan Province China; 3 West China Clinical Skills Training Center, West China School of Medicine/West China Hospital, Sichuan University, 37 Guoxue Xiang, Sichuan Province 610041 Chengdu, China

**Keywords:** myopic prevalence, spherical equivalent error, axial length, corneal radius, axial length/corneal radius, Chinese children

## Abstract

**Background:**

The prevalence of myopia among children in Chengdu is unknown. The aim of this study was to determine the prevalence of myopia in 3- to 14-year-old Chinese children in Chengdu.

**Methods:**

This study was a school-based cross-sectional study in children aged 3–14 years. Visual acuity (VA), spherical equivalent error (SER) with noncycloplegic autorefraction, axial length (AL) and corneal radius (CR) were measured.

**Results:**

A total of 19,455 children were recruited for this study. The prevalence of myopia was 38.1 %; the prevalence of low myopia was 26.6 %, that of moderate myopia was 9.8 %, and that of high myopia was 1.7 %. The prevalence of myopia and SER increased with age from 6 years old. The prevalence of myopia was higher, and the SER indicated more severe myopia in the girls than in the boys (40.1 % vs. 36.2 %, χ^2^ = 30.67, d_f_ = 1, *P* < 0.001; -0.93 D ± 1.75 D vs. -0.84 D ± 1.74 D, t = 3.613, d_f_=19,453, *P* < 0.001). The girls had a higher prevalence of myopia and myopic SER than did the boys aged 9 years and older (*P* < 0.05). Among the myopic children, the rates of uncorrected, undercorrected and fully corrected myopia were 54.8 %, 31.1 and 14.1 %, respectively. AL and AL/CR increased with age from 6 years old, but CR remained stable after 4 years old. The AL was longer, and the CR was flatter in the boys than in the girls aged 3 to 14 years old (*P* < 0.05).

**Conclusions:**

The prevalence of myopia, AL and AL/CR increased, and the SER became more myopic with age from 6 years old. The girls had a higher prevalence of myopia and myopic SER than did the boys, but the boys had a longer AL, flatter CR and higher AL/CR ratio than did the girls. The rate of uncorrected myopia was very high in the myopic children. More actions need to be taken to decrease the prevalence of myopia, especially uncorrected myopia in children.

## Background

Myopia has become the most common type of ametropia worldwide. Myopia, especially high myopia, is one of the main causes of visual impairment [1–3]. Myopia affected nearly 30 % of the total population in the early 21st century, but this rate is increasing [4, 5]. By 2050, the prevalence of myopia is expected to be nearly 50 %, and that of high myopia is expected to reach 10 % [6]. Myopia is characterized by a high incidence, early onset and rapid progression in East Asia. The rapid development of myopia needs to be considered because it not only leads to inconvenience in daily life but also causes ocular diseases, such as macular disease, retinal detachment, and glaucoma. These conditions cause very large social and economic burdens [7, 8]. Therefore, myopia is of great concern, especially in East Asia. While the prevalence of myopia among children in some large cities in China has been reported [9–12], the prevalence of myopia among children in Chengdu has not been determined, even though Chengdu is an important central city in Western China. Children usually develop myopia at the age of 6 [13], so myopic prevention and control is needed in younger children. At present, there are a few studies on the prevalence of myopia in children aged 3 years and older. Because the age at which children begin school varies, it is better to determine stratify the prevalence of myopia by age in children.

Therefore, the purpose of this study was to investigate the prevalence of myopia in children aged 3 to 14 years in Chengdu. The results will be helpful for myopia prevention and health policy planning.

## Methods

### Subjects

This study was conducted in accordance with the Declaration of Helsinki and approved by the ethics committee of the hospital [No. 2019 review (90)]. The purpose and procedures were explained to the subjects and their guardians, who signed informed consent forms before commencing this study.

This cross-sectional study was conducted from June to December 2019. The participating children were students at kindergartens, primary schools and junior high schools in Chengdu, Sichuan Province, China.

### Procedures

Visual acuity (VA) and spherical equivalent error (SER) with noncycloplegic autorefraction were determined by two experienced senior optometrists. Each eye was measured at least three times with a Topcon KR800 (Topcon Co., Tokyo, Japan). Repeated measurements were taken if any measured value deviated from the other two values by more than 0.50 dioptres (D). Finally, three reliable measurements were averaged for analysis.

VA with and without habitual spectacles (if available) was measured for each eye in a well-lit room during the daytime at a distance of five metres. A standard tumbling E logMAR chart was used. VA was determined by the line with the smallest letter size where the child could correctly identify the orientation of more than half of the letters. The logMAR scores were then recorded for each child.

Ocular biometry parameters, axial length (AL) and corneal radius (CR), were measured with a LENSTAR LS900 (Haag-Streit AG, Switzerland). AL was the distance between the anterior corneal vertex and the retinal pigment epithelium with fixation. CR was measured along the flattest and steepest meridians, and the mean CR was used for analysis. The AL/CR ratio was calculated by dividing AL by the mean CR. Because there is a strong correlation between the two eyes [14], only the data for the right eye were used for analysis.

### Definitions

As suggested by Thorn et al. [14], combining non-cycloplegic refraction with visual acuity makes the judgment of myopia more accurate than by non-cycloplegic refraction alone. Hence, myopia was defined as SER ≤ − 0.50 D [15] + uncorrected VA (UCVA) > 0.3 logMAR for children aged 3 years, > 0.2 logMAR for children aged 4 to 5 years, and > 0 logMAR for children aged 6 years or older. The myopia cases were categorized by severity as follows: low myopia (-3.00 D ≤ SER ≤ -0.50 D), moderate myopia (-6.00 D < SER < -3.00 D), and high myopia (SER ≤ -6.00 D).

If a child was judged as having myopia without correction, he or she was considered to have uncorrected myopia. If the case of myopia was corrected with spectacles and VA was normal (≤ 0.3 logMAR for age 3, ≤ 0.2 logMAR for ages 4 to 5, and ≤ 0 logMAR for ages 6 and higher), it was defined as fully corrected myopia; otherwise, it was regarded as undercorrected myopia.

### Statistical analysis

Statistical analysis was performed using SPSS 20.0 (IBMSPSS, Chicago, IL, USA). Chi-squared tests were used to evaluate the prevalence of myopia.

If the data were normally distributed, an independent t test was used to compare the means of two groups. The statistical tests were two-sided, and *P* < 0.05 was considered statistically significant. One-way ANOVA was used to assess the significance of the differences among no fewer than three groups. If *P* < 0.05, post hoc LSD was used to compare two groups. If the data were not normally distributed, a nonparametric test was used.

## Results

### Subject characteristics

A total of 19,455 children finally took part in the study; 10,118 were boys, accounting for 52.0 % of the study population, and 9337 were female, accounting for 48.0 % of the study population. The mean ± SD age was 9.18 ± 2.50 years old.

### Prevalence of myopia

The overall prevalence of myopia was 38.1 % (7415). The total prevalence of myopia and the prevalence of myopia stratified by sex and age are shown in Table 1. The total prevalence of myopia remained stable between ages 3 and 5 years (3 vs. 4 years *P* = 0.88, 3 vs. 5 years *P* = 0.968, 4 vs. 5 years *P* = 0.831) but increased from ages 6 to 13 years (all *P* < 0.001). Although the children aged 14 years (74.9 %) had a higher prevalence of myopia than did the children aged 13 years (72.1 %), the difference was not significant (*P* = 0.13).

**Table 1:** Total prevalence of myopia and prevalence of myopia stratified by sex and age

Age (y)	Prevalence of Myopia (N_1_/N_2_)			χ^2^	*P* value
	Total	Boys	Girls		
3	1.9 % (3/159)	1.2 % (1/80)	2.5 %(2/79)	0.353	0.553
4	2.6 % (6/235)	3.6 % (5/139)	1.0 % (1/96)	1.49	0.22
5	1.7 % (4/234)	2.3 % (3/128)	0.9 % (1/106)	0.692	0.406
6	8.6 % (199/2304)	7.8 % (92/1173)	9.5 % (107/1131)	1.91	0.167
7	14.5 % (427/2936)	15.1 % (237/1569)	13.9 % (190/1367)	3.447	0.07
8	25.9 % (678/2621)	25.3 % (346/1365)	26.4 % (332/1256)	0.402	0.526
9	37.2 % (904/2431)	34.7 % (432/1245)	39.8 % (472/1186)	6.76	0.009*
10	48.7 % (1138/2336)	45.9 % (546/1190)	51.7 % (592/1146)	7.795	0.005*
11	56.5 % (1248/2210)	53.4 % (611/1145)	59.8 % (637/1065)	9.338	0.002*
12	66.2 % (1075/1623)	62.2 % (532/855)	70.7 % (543/768)	13.0	< 0.001**
13	72.1 % (1031/1429)	69.1 % (509/737)	75.4 % (522/692)	7.21	0.007*
14	74.9 % (702/937)	71.1 % (350/492)	79.1 % (352/445)	7.89	0.005*

N: number, N1: the number of myopic children, N2: the total number of children, * represents *P* < 0.05, ** represents *P* < 0.001

The prevalence of myopia in the girls (40.1 %) was higher than that in the boys (36.2 %) (χ^2^ = 32.30, d_f_ = 1, *P* < 0.001). From age 9, the prevalence of myopia in the girls was higher than that in the boys (*P* < 0.05).

**Fig. 1:**
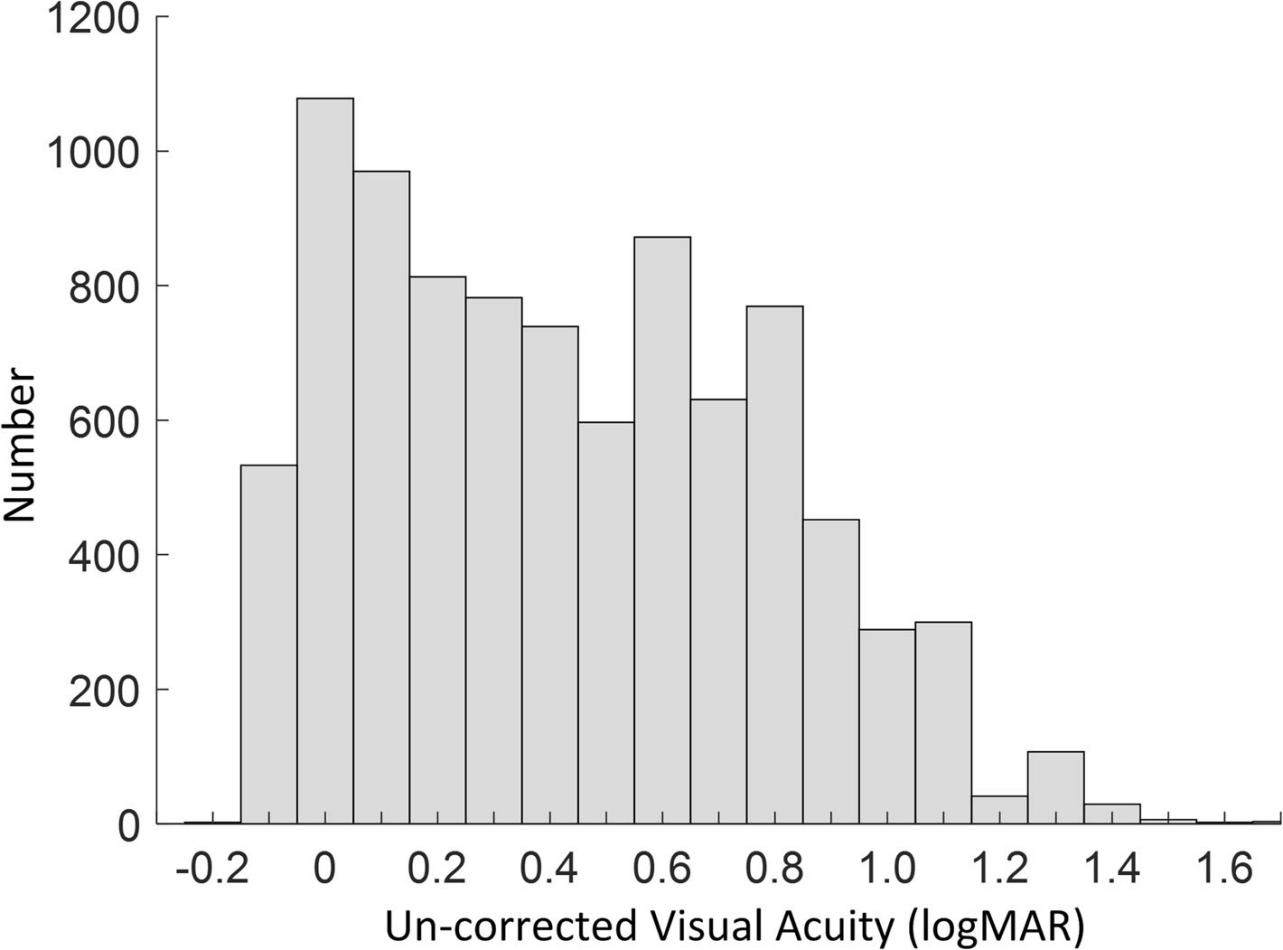
Histogram of the UCVA values of all children aged 6 years or older with SER ≤ − 0.50 D

We further analysed the distribution of UCVA values to demonstrate the impact of these thresholds in myopia definition. Figure 1 shows the histogram of the UCVA values of all children aged 6 years or older with SER ≤ − 0.50 D. Taking children aged 6 years or older as example, comparing with defining myopia with only SER,adding UCVA (with > 0 logMAR for children aged 6 years or older) reduced 1613 myopia children in this paper. From the histogram, the number of children defined as myopia will be reduced by hundreds when the UCVA threshold is raised by 0.1. It should be noted that we just analysed the UCVA on children aged 6 years or older because the number of children aged 3–5 years is small.

The severity categories of myopia stratified by age are shown in Table 2. Low myopia (26.6 %) had the highest prevalence, followed by moderate myopia (9.8 %) and high myopia (1.7 %) (low vs. moderate χ^2^ = 1838.0, d_f_=1, *P* < 0.001; low vs. high χ^2^ = 4976.6, d_f_=1, *P* < 0.001; moderate vs. high χ^2^ = 1190.5, d_f_=1, *P* < 0.001). The prevalence of low myopia in the girls was higher than that in the boys (28.4 % vs. 25.0 %, χ^2^ = 29.02, d_f_=1, *P* < 0.001), while the prevalence of moderate myopia (9.5 % vs. 10.1 %, χ^2^ = 2.01, d_f_=1, *P* = 0.157) and high myopia (1.7 % vs. 1.7 %, χ^2^ = 0.108, d_f_=1, *P* = 0.743) did not significantly differ between the girls and boys.

**Table 2:** The percentage of children with different severities of myopia stratified by age

Age (N)	Myopia Categories (N_1_)		
	Low	Moderate	High
3 (159)	1.9 % (3)	0.0 % (0)	0.0 % (0)
4 (235)	1.7 % (4)	0.9 % (2)	0.0 % (0)
5 (234)	1.3 % (3)	0.4 % (1)	0.0 % (0)
6 (2304)	8.2 % (189)	0.3 % (7)	0.1 % (3)
7 (2936)	13.5 % (395)	0.9 % (27)	0.2 % (5)
8 (2621)	23.0 % (602)	2.6 % (68)	0.3 % (8)
9 (2431)	31.3 % (762)	5.5 % (133)	0.4 % (9)
10 (2336)	36.3 % (849)	11.4 % (266)	1.0 % (23)
11 (2210)	38.6 % (854)	15.9 % (351)	1.9 % (43)
12 (1623)	39.1 % (634)	23.1 % (375)	4.1 % (66)
13 (1429)	39.2 % (560)	26.7 % (382)	6.2 % (89)
14 (937)	34.3 % (321)	32.1 % (301)	8.5 % (80)

N: number, N1: the number of myopic children

### Correction of myopia

Among 7415 cases of myopia, 54.8 % (4062) were uncorrected, 31.1 % (2306) were undercorrected, and only 14.1 % (1047) were fully corrected. The percentages of uncorrected, undercorrected and fully corrected cases in the boys and girls were 55.8 and 53.8 % (χ^2^ = 3.12, d_f_=1, *P* = 0.078), 29.7 and 32.5 % (χ^2^ = 6.67, d_f_=1, *P* = 0.0098), and 14.5 and 13.8 % (χ^2^ = 0.83, d_f_=1, *P* = 0.36), respectively. Hence, there were no significant differences between the boys and girls in the percentages of uncorrected and fully corrected myopia, but the undercorrected rate was slightly higher in girls than boys.

**Fig. 2:**
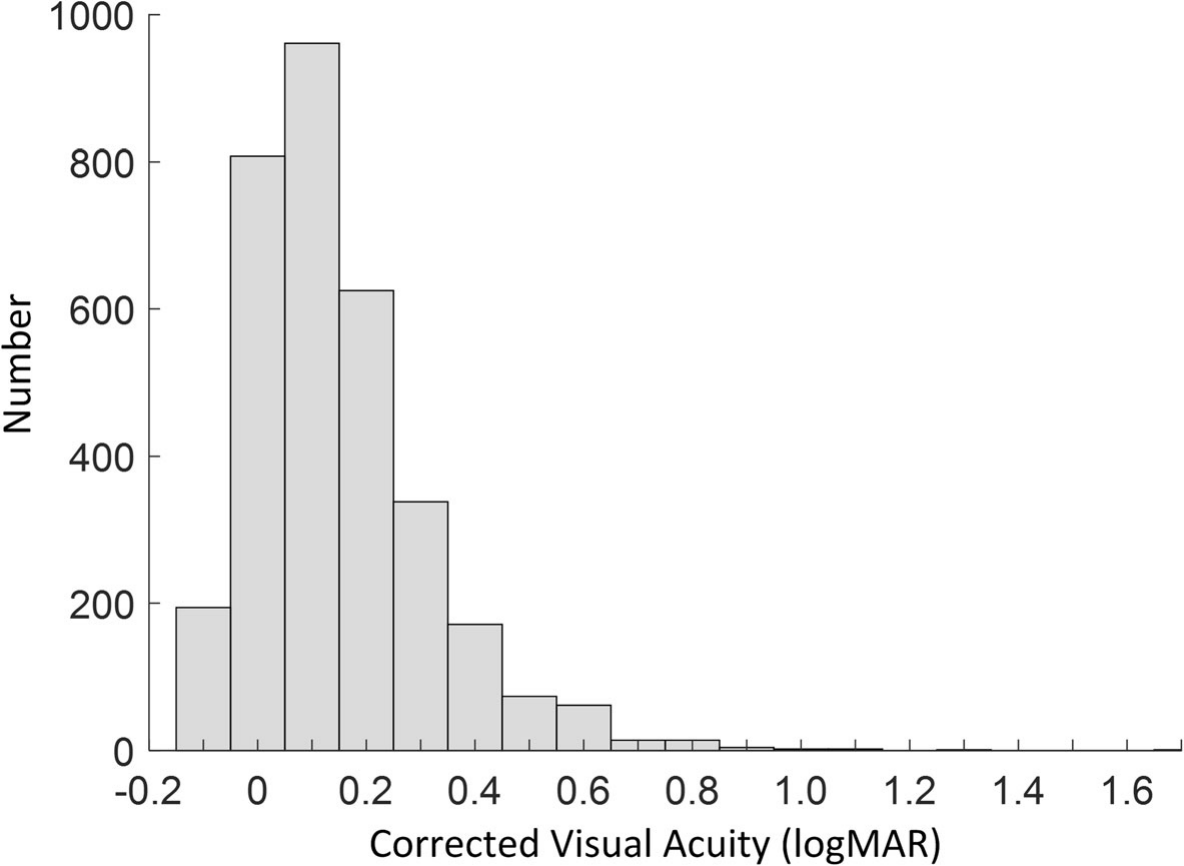
Histogram of corrected visual acuity of all myopic children aged 6 years or older

To specifically describe the distribution of the undercorrection, we added the histogram of corrected visual acuity of all myopic children aged 6 years or older. Figure 2 showed that most myopic children were not corrected to ≤ 0 logMAR (i.e., undercorrection with the definition in this paper). Figure 2 also showed the overall distribution of corrected visual acuity after correction, which provided more information for the evaluation of visual correction.

### Spherical equivalent refraction

The mean ± SD SER was − 0.88 ± 1.74 D, with a minimum of -14.38D and a maximum of + 8.50D. The total SER and SER values stratified by sex and age are shown in Table 3.

**Table 3:** Total SER and SER values stratified by sex and age

Age (N)	SER (D)			t	*P* value
	Total	Boys	Girls		
3 (159)	+ 0.32 ± 1.01	+ 0.22 ± 1.29	+ 0.42 ± 0.60	-1.21	0.228
4 (235)	+ 0.22 ± 0.82	+ 0.17 ± 0.85	+ 0.29 ± 0.76	-1.17	0.243
5 (234)	+ 0.37 ± 0.54	+ 0.35 ± 0.60	+ 0.39 ± 0.45	-0.514	0.608
6 (2304)	+ 0.22 ± 0.89	+ 0.22 ± 1.03	+ 0.21 ± 0.73	0.315	0.753
7 (2936)	-0.03 ± 0.95	-0.04 ± 0.92	-0.01 ± 0.98	-0.733	0.463
8 (2621)	-0.40 ± 1.14	-0.40 ± 1.21	-0.39 ± 1.06	-0.275	0.783
9 (2431)	-0.71 ± 1.31	-0.65 ± 1.33	-0.76 ± 1.29	2.072	0.038*
10 (2336)	-1.13 ± 1.59	-1.07 ± 1.52)	-1.20 ± 1.66	2.088	0.037*
11 (2210)	-1.48 ± 1.79	-1.41 ± 1.83	-1.56 ± 1.74	2.048	0.041*
12 (1623)	-1.96 ± 2.07	-1.85 ± 2.08	-2.07 ± 2.05	2.118	0.034*
13 (1429)	-2.36 ± 2.10	-2.30 ± 2.11	-2.43 ± 2.10	1.11	0.267
14 (937)	-2.71 ± 2.32	-2.53 ± 2.35	-2.90 ± 2.26	2.398	0.017*

N: number, D: dioptres, * represents *P* < 0.05, **represents *P* < 0.001

There were no significant differences in SER between the 3-6-year-olds (3 vs. 4 years *P* = 0.506, 3 vs. 5 years *P* = 0.744, 3 vs. 6 years *P* = 0.403, 4 vs. 5 years *P* = 0.27, 5 vs. 6 years *P* = 0.136), but SER increased with age from ages 7 to 14 years (*P* < 0.001).

**Fig. 3:**
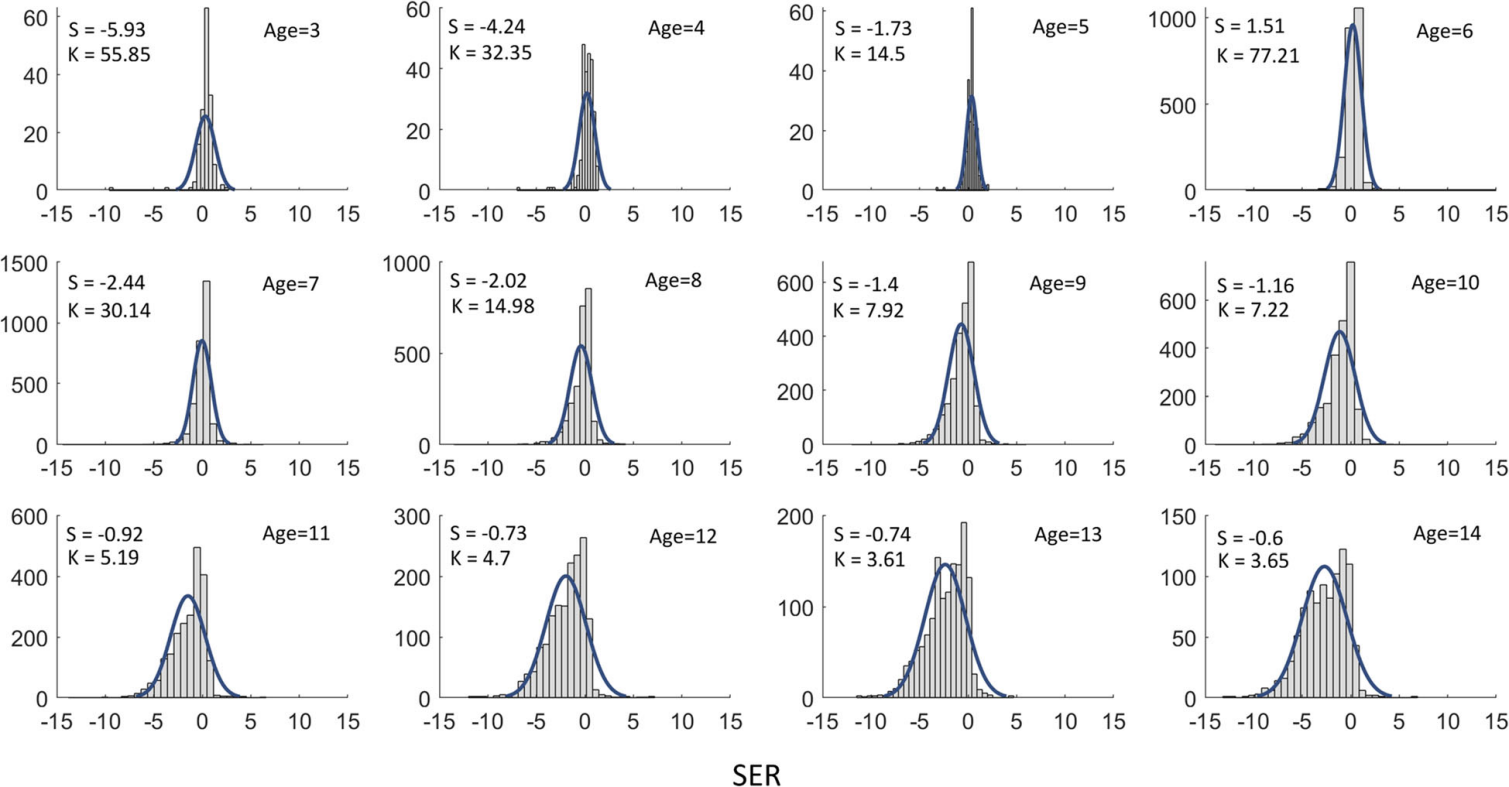
Histograms of the SER by age with the Skew (S), Kurtosis (K) and Gaussian fitting curves were displayed in each sub-figure

The mean ± SD SER was − 0.84 ± 1.74 D in the boys and − 0.93 ± 1.75 D in the girls, and the SER in the girls was more myopic than that in the boys (t = 3.61, d_f_ = 19,453, *P* < 0.001). Moreover, from ages 9 to 14 years, the SER in the girls was more myopic than was that in the boys, except for at age 13 years, even though the trend still existed at this age (see Table 3). To demonstrate the distribution shape of large populations, the histograms of the data for SER by age were shown with skew and kurtosis in Fig. 3.

### Axial length

The maximum AL was 29.72 mm, the minimum AL was 19.58 mm, and the mean ± SD was 23.53 ± 1.14 mm. The total AL and AL values stratified by sex and age are shown in Table 4.

**Table 4:** Total AL and AL values stratified by sex and age

Age (N)	AL (mm)			t	*P* value
	Total	Boys	Girls		
3 (159)	21.93 ± 0.62	22.15 ± 0.60	21.71 ± 0.56	4.852	< 0.001**
4 (235)	22.33 ± 0.65	22.55 ± 0.63	22.00 ± 0.53	6.972	< 0.001**
5 (234)	22.41 ± 0.66	22.68 ± 0.60	22.09 ± 0.59	7.628	< 0.001**
6 (2304)	22.64 ± 0.72	22.90 ± 0.71	22.37 ± 0.63	18.993	< 0.001**
7 (2936)	22.95 ± 0.77	23.21 ± 0.73	22.65 ± 0.71	21.086	< 0.001**
8 (2621)	23.25 ± 0.83	23.51 ± 0.79	22.97 ± 0.78	17.824	< 0.001**
9 (2431)	23.54 ± 0.91	23.79 ± 0.86	23.28 ± 0.89	14.261	< 0.001**
10 (2336)	23.84 ± 1.01	24.10 ± 0.96	23.57 ± 0.98	13.076	< 0.001**
11 (2210)	24.05 ± 1.05	24.29 ± 1.04	23.78 ± 1.00	11.666	< 0.001**
12 (1623)	24.24 ± 1.12	24.46 ± 1.15	23.99 ± 1.03	8.615	< 0.001**
13 (1429)	24.40 ± 1.14	24.69 ± 1.12	24.09 ± 1.08	10.408	< 0.001**
14 (937)	24.64 ± 1.25	24.86 ± 1.28	24.40 ± 1.16	5.808	< 0.001**

N: number, mm: millimetres, **represented P < 0.001

**Fig. 4:**
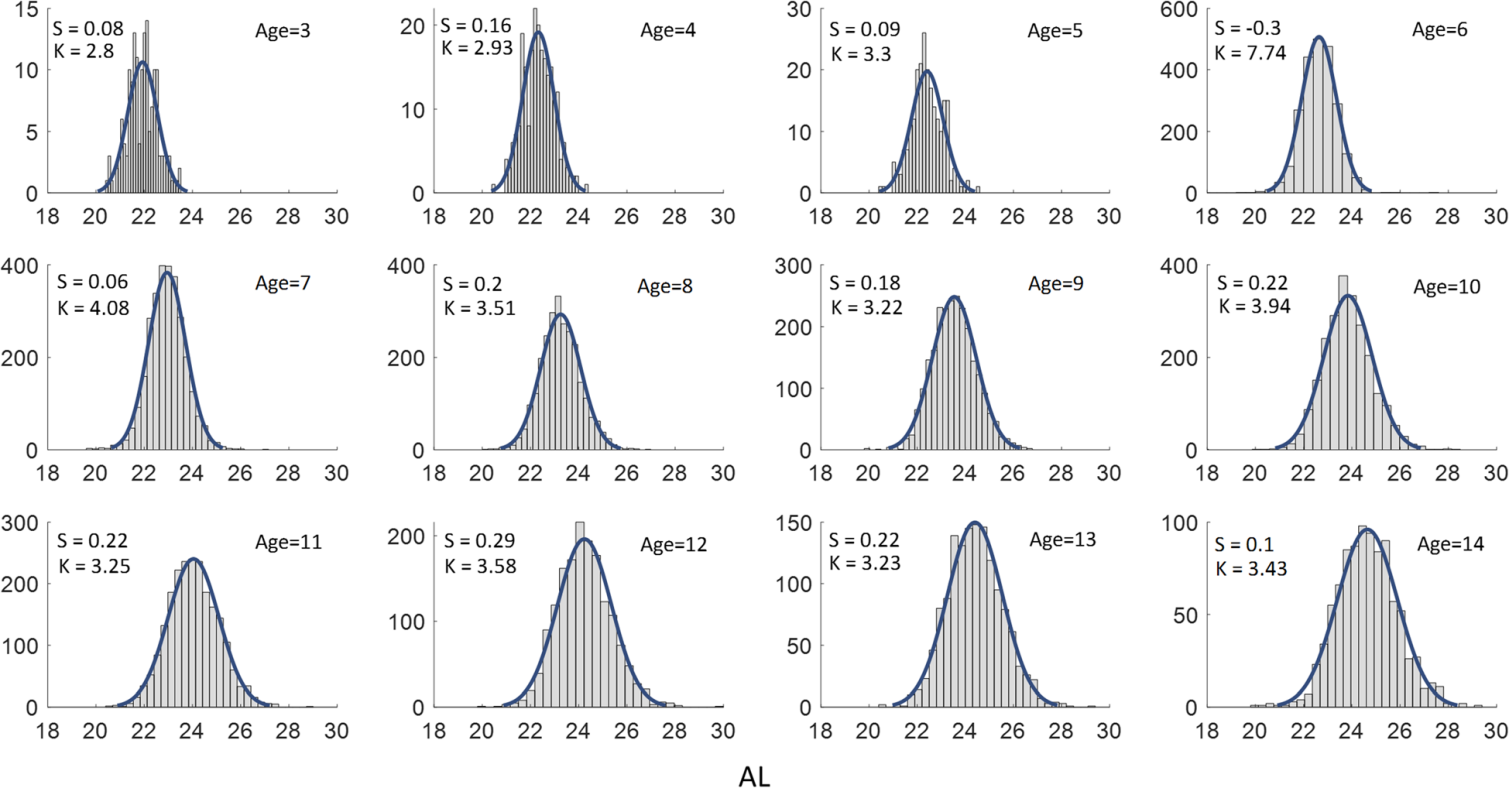
Histograms of the AL by age with the skew (S), kurtosis (K) and Gaussian fitting curves were displayed in each sub-figure

AL increased with age (*P* < 0.001), except between ages 4 and 5 years (*P* = 0.321). The AL in the boys (23.78 mm ± 1.12 mm) was longer than that in the girls (23.26 mm ± 1.09 mm) (t = 23.94, d_f_=19,453, *P* < 0.001). From ages 3 to 14 years, the boys had longer ALs than did the girls (see Table 4). The histograms of the data for axial length (AL) by age were shown with skew and kurtosis in Fig. 4.

### Corneal radius

The maximum CR was 9.04 mm, the minimum was 6.42 mm, and the mean ± SD was 7.84 ± 0.26 mm. The total CR and CR values stratified by sex and age are shown in Table 5.

**Table 5:** Total CR and CR values stratified by sex and age

Age (N)	CR (mm)			t	*P* value
	Total	Boys	Girls		
3 (159)	7.78 ± 0.25	7.84 ± 0.25	7.72 ± 0.25	3.099	0.002*
4 (235)	7.85 ± 0.25	7.91 ± 0.26	7.77 ± 0.22	4.225	< 0.001**
5 (234)	7.84 ± 0.27	7.92 ± 0.26	7.75 ± 0.24	5.154	< 0.001**
6 (2304)	7.83 ± 0.26	7.89 ± 0.26	7.76 ± 0.24	12.318	< 0.001**
7 (2936)	7.84 ± 0.26	7.90 ± 0.25	7.76 ± 0.24	15.401	< 0.001**
8 (2621)	7.84 ± 0.26	7.90 ± 0.25	7.77 ± 0.24	12.965	< 0.001**
9 (2431)	7.84 ± 0.26	7.92 ± 0.26	7.77 ± 0.25	14.103	< 0.001**
10 (2336)	7.85 ± 0.27	7.92 ± 0.26	7.78 ± 0.25	13.089	< 0.001**
11 (2210)	7.85 ± 0.26	7.91 ± 0.25	7.78 ± 0.25	12.067	< 0.001**
12 (1623)	7.84 ± 0.26	7.90 ± 0.25	7.78 ± 0.25	9.702	< 0.001**
13 (1429)	7.83 ± 0.26	7.90 ± 0.25	7.76 ± 0.25	11.402	< 0.001**
14 (937)	7.85 ± 0.25	7.91 ± 0.24	7.78 ± 0.24	8.104	< 0.001**

N: number, mm: millimetres, * represents *P* < 0.05, **represents *P* < 0.001

The CR of the 3-year-old children was steeper than that of the children of other ages (*P* < 0.05) and tended to be stable from the age of 4 years.

The mean ± SD CR was 7.91 ± 0.25 mm in the boys and 7.77 ± 0.25 mm in the girls. The CR in the boys was flatter than that in the girls (t = 37.44, d_f_=19,453, *P* < 0.001). From the age of 3 to 14 years, the CR in the boys was flatter than that in the girls (see Table 5).

### Axial length/corneal radius

The maximum AL/CR was 3.72, the minimum was 2.43, and the mean ± SD was 3.00 ± 0.13. The total AL/CR and AL/CR values stratified by sex and age are shown in Table 6.

**Table 6:** Total AL/CR and AL/CR values stratified by sex and age

Age (N)	AL/CR			t	*P* value
	Total	Boys	Girls		
3 (159)	2.82 ± 0.06	2.83 ± 0.06	2.81 ± 0.06	1.453	0.148
4 (235)	2.84 ± 0.06	2.85 ± 0.06	2.83 ± 0.06	2.602	0.01*
5 (234)	2.86 ± 0.07	2.86 ± 0.07	2.85 ± 0.07	1.634	0.104
6 (2304)	2.89 ± 0.07	2.90 ± 0.08	2.88 ± 0.07	7.053	< 0.001**
7 (2936)	2.93 ± 0.08	2.94 ± 0.08	2.92 ± 0.08	6.39	< 0.001**
8 (2621)	2.97 ± 0.09	2.98 ± 0.09	2.95 ± 0.09	6.239	< 0.001**
9 (2431)	3.00 ± 0.10	3.01 ± 0.10	3.00 ± 0.11	2.267	0.023*
10 (2336)	3.04 ± 0.11	3.04 ± 0.11	3.03 ± 0.12	2.77	0.006*
11 (2210)	3.06 ± 0.13	3.07 ± 0.13	3.06 ± 0.12	2.682	0.007*
12 (1623)	3.09 ± 0.13	3.10 ± 0.13	3.09 ± 0.13	2.121	0.008*
13 (1429)	3.11 ± 0.14	3.12 ± 0.14	3.10 ± 0.14	2.778	0.006*
14 (937)	3.14 ± 0.15	3.14 ± 0.15	3.13 ± 0.15	0.794	0.427

N: number, * represents *P* < 0.05, **represents *P* < 0.001

AL/CR increased with age (*P* < 0.001), except between ages 4 and 5 years (*P* = 0.168), which was consistent with the change in AL.

**Fig. 5:**
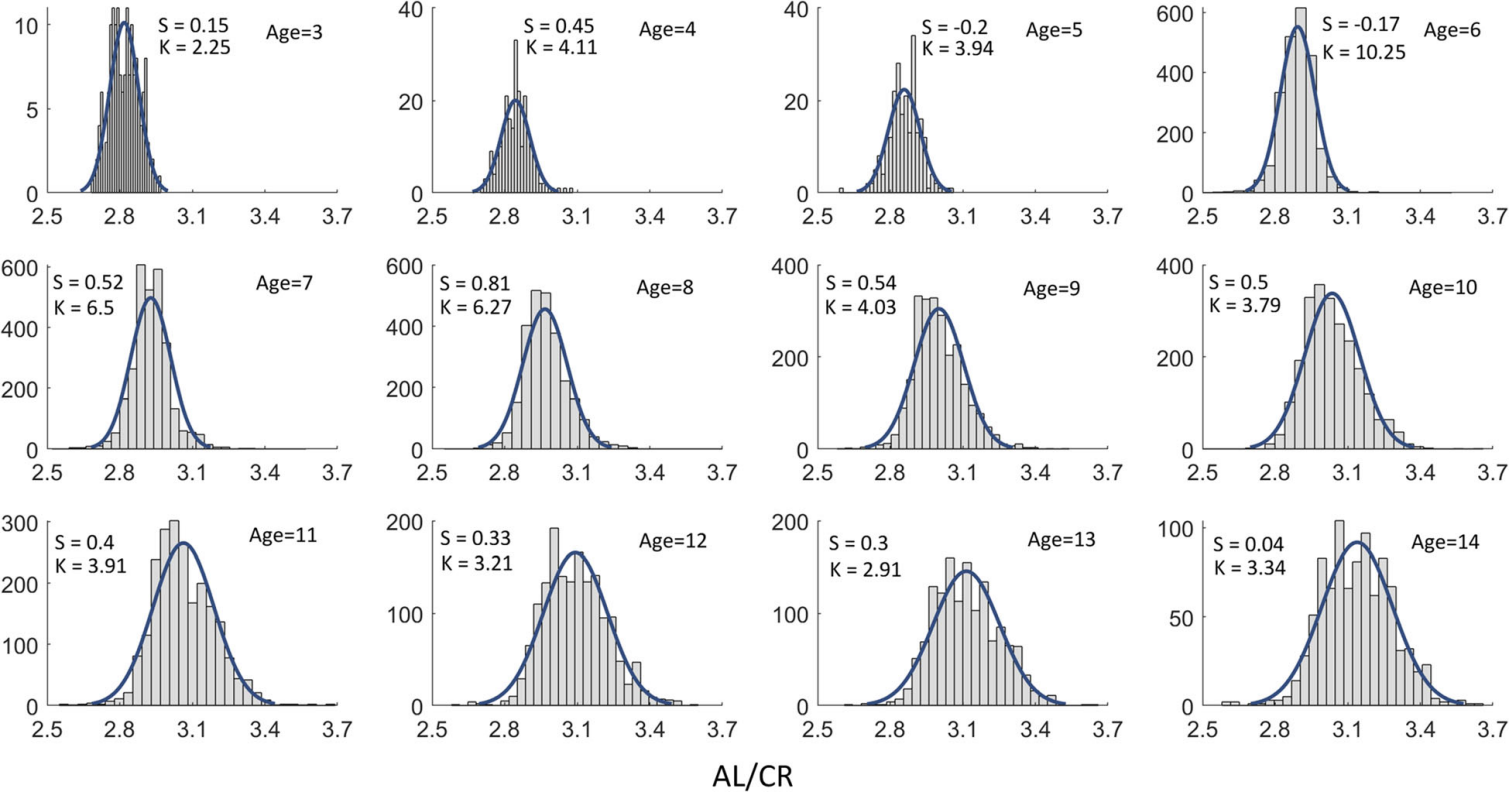
Histograms of the AL/CR by age with the skew (S), kurtosis (K) and Gaussian fitting curves were displayed in each sub-figure

The AL/CR ratio of the boys (3.01 ± 0.13) was higher than that of the girls (2.99 ± 0.13) (t = 8.07, d_f_=19,453, *P* < 0.001). The AL/CR of the boys was always higher than that of the girls, except for at ages 3, 5 and 14 years, even though the trend still existed at these ages (see Table 6). The histograms of the data for AL/CR by age were shown with skew and kurtosis in Fig. 5.

**Fig. 6:**
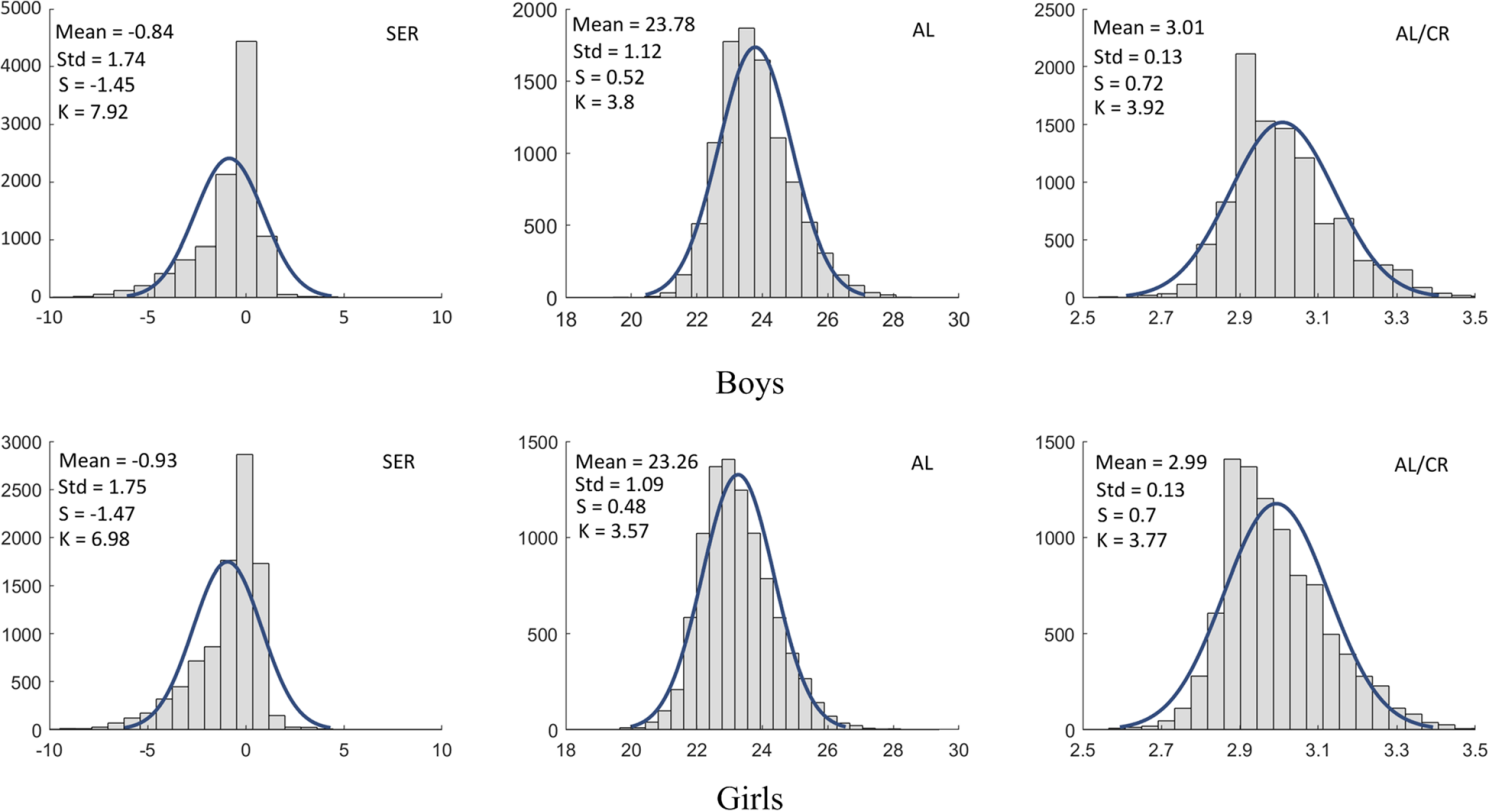
Histograms of the SER, AL, and AL/CR by gender with the mean, std, skew (S), kurtosis (K) and Gaussian fitting curves were displayed in each sub-figure

**Fig. 7:**
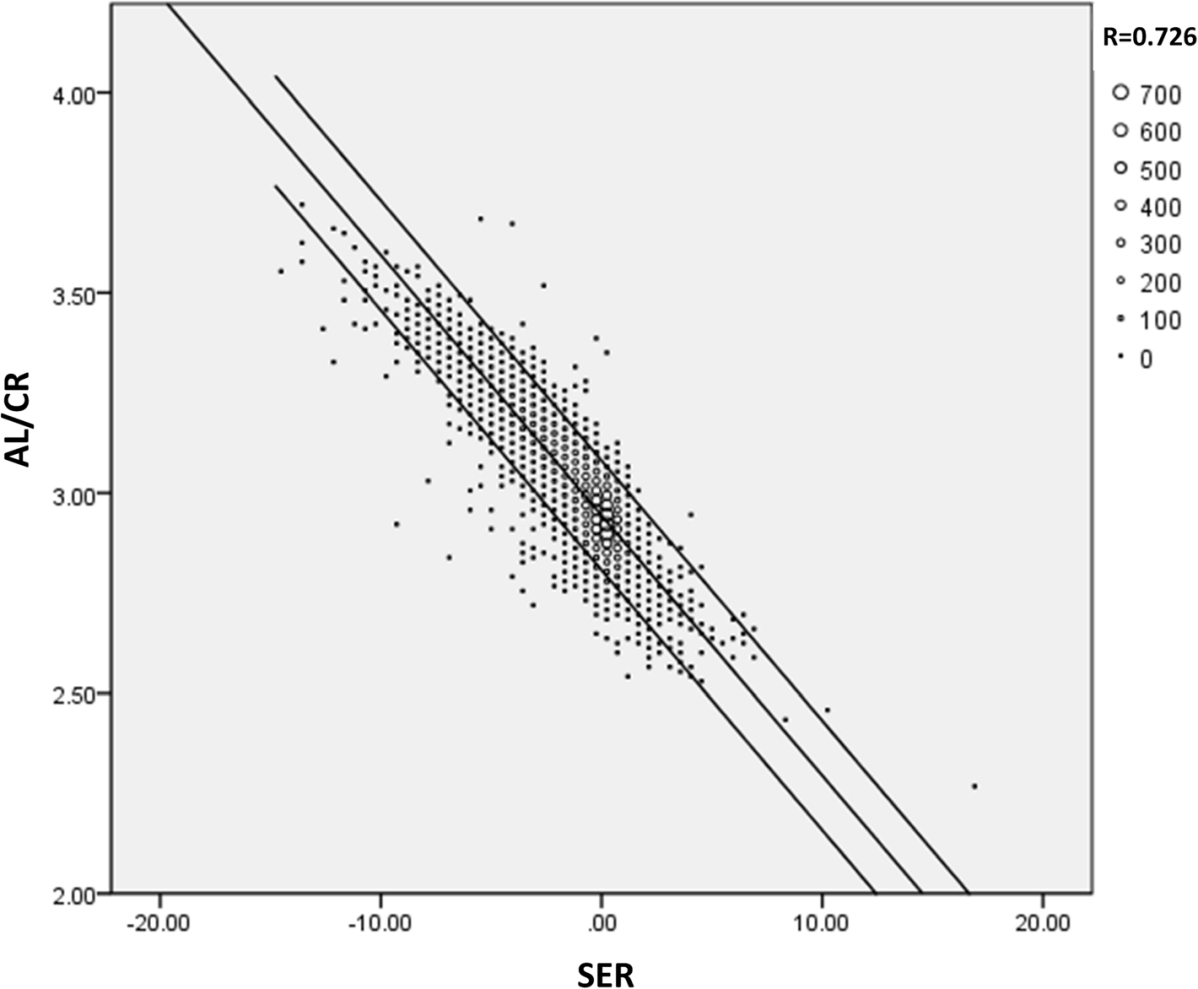
The correlation between AL/CR and SER. The linear correlation coefficient was 0.726. Different size of dot represented various number of children

In addition, histograms of the SER, AL, and AL/CR by gender were shown in Fig. 6. Considering the highly correlation between AL/CR and SER, a fitting plot was provided in Fig. 7, which showed the linear correlation coefficient between AL/CR and SER was 0.726.

## Discussion

Information about the prevalence of myopia is very important for health policy planning [16]. Although longitudinal cohort research is ideal, this kind of research needs a considerable amount of resources, so it is not commonly performed. Cross-sectional studies are also useful when myopia is assessed by age. Therefore, this cross-sectional study investigated the prevalence of myopia in children aged 3 to 14 years. The data in the study were comprehensive, as vision, autorefraction, AL and AL/CR results were assessed. This study had a large sample size. In addition, the extent of myopic correction was also investigated.

In this study, myopia was assessed by both noncycloplegic autorefraction (SER ≤ − 0.50 D) and visual acuity at different ages. On the one hand, this method ensures high participation and reduces the side effects of cycloplegia. On the other hand, the additional consideration of vision at different ages makes the judgement of myopia more accurate.

Most of the studies from China gave the prevalence of myopia for an age range and used non-cycloplegic refraction. For example, Wang et al. reported the prevalence rate of myopia among 4801 children with a mean age of 12.3 years in eastern China, 63.1 % had myopia and 9.4 % had high myopia [17]. The study in Taiwan showed the prevalence of myopia and high myopia of students from Grade 1–9 were 42.0 and 2.0 %, respectively [18]. Another study in Hong Kong revealed that the overall prevalence of myopia from Grade 1 to Grade 6 was 37.7 % [19]. Similarly, all participants in our study underwent non-cycloplegic refraction and the overall prevalence of myopia was 38.1 % in 3-14-year-old children. Considering that the 3-14-year-old children roughly in kindergarten to Grade 7 and with a mean age of 9.2 years in this study, the overall prevalence was similar to Choy ’s [19], but lower than Wang’s [17] and Lai’s [18] because of younger participants in this study. All these studies revealed a statistically significant increase in the myopic prevalence with increasing age.

However, in this study, we provided age-specific results that indicated more details of myopic prevalence. For example, Wang et al. reported the baseline prevalence of myopia in grade 1 was 12.0 and 67.4 % in grade 7 in regions of East and Southeast Asia [13]. Generally speaking, most of the students in grade 1 were 6- or 7-year-old. Our study showed the prevalence of myopia was 8.6 and 14.5 % at the age of 6 and 7 specifically. And the overall prevalence of myopia was 11.9 % for all 6- and 7-year-old children, which was close to the baseline prevalence of myopia in grade 1 [13]. Besides, our age-specific results revealed a significantly higher prevalence of myopia in 7-year-old children than that in 6-year-old children. Age-specific prevalence values could provide some basis for further study.

On the other hand, some studies also measured cycloplegic refraction, which lead to a more accurate estimate of the prevalence of myopia. For example, Guo et al. reported the prevalence of myopia in China among the 1127 children with cycloplegic refraction [20]. Their study showed the prevalence of myopia was 0 % at the age of 3 years and 3.7 % at the age of 6 years. Comparatively, the prevalence of myopia was 1.9 % at the age of 3 years and 8.6 % at the age of 6 years in our study using non-cycloplegic refraction. This result verified again that non-cycloplegic refraction would overestimate the prevalence of myopia, about 5 % with a rough overestimation from 6-year-old children from different studies. This is similar to the reported overestimation of the prevalence of myopia about 7 % with non-cycloplegic refraction [21].The study showed that the prevalence of myopia remained stable before the age of 6 years but increased with age thereafter (8.6 % at 6 years old and 74.9 % at 14 years old). This finding is consistent with that of Wang [22]. The prevalence of myopia of the children aged 14 years (74.9 %) was slightly higher than that of the children aged 13 years (72.1 %), but the difference was not significant. The reason for the nonsignificant finding may be the small sample size, and more studies are needed in the future.

The total prevalence of myopia in the girls was higher than that in the boys. This finding is consistent with those in previous studies [14, 23, 24]. This may be because girls spend more time studying and less time participating in outdoor activities than do boys. We also found that the prevalence of myopia in the girls was always higher than that in the boys aged 9 years and older. Thorn et al. found that the prevalence of myopia was higher for girls than for boys from grade 5 in primary school. This finding is consistent with ours because children in grade 5 are close to 9 years old [14]. In regard to myopia severity, the prevalence of low myopia was the highest and that of high myopia was the lowest, which is consistent with Xie’s findings [12]. In addition, the prevalence of low myopia in the girls was higher than that in the boys, but the prevalence of moderate myopia and high myopia was similar between the girls and boys. That is, the total prevalence of myopia in the girls was higher than that in the boys, and this difference was mainly determined by the cases of low myopia. This finding was opposite to Li, who observed that the prevalence of moderate and high myopia were higher in girls than in boys [23]. The discrepancy may be that the majority of children in our cross-sectional study were primary school students, but Li et al. performed a retrospective, longitudinal cohort study in junior high schools over 10 years.

Our study still showed that the prevalence rates of myopia and high myopia were 8.6 and 0.1 %, respectively, at the age of 6 (nearly grade 1) but were as high as 66.1 and 4.0 % at the age of 12 (nearly grade 7). Wang et al. found that the prevalence of myopia was 12 %, and the high myopia rate was less than 1 % in grade 1, but these rates were as high as 67.4 and 2 % in grade 7. Our results are consistent with Wang’s results [13].

Among the myopic children, we still found that more than half were uncorrected, and less than 30 % were fully corrected. This may be a common phenomenon. Hu et al. [25] reported that the spectacle-wearing rate was nearly 5.0 % in Yunnan minority children, 6.2 % in Yunnan Han children, and 15.3 % in Guangzhou Han children aged 9 to 12 years old. Even in Hong Kong, less than a quarter of the parents knew their children had refractive errors, and less than 20 % of the children wore glasses [19]. Our results showed that the ratios of uncorrectedand fully corrected myopia were similar between the boys and girls. This is to say, the parents do not address their children’s myopic conditions sufficiently. In addition, some children are too afraid of being criticized by their parents to tell them when they notice worsened vision, and some parents are not willing to let their children wear glasses. Therefore, great effort is still needed to increase awareness on myopia prevention and control.In addition, the uncorrected rate of myopia may be overestimated when conducted with noncycloplegic autorefraction. Besides, we also found that the undercorrected rate was higher in girls than boys, which maybe that the myopic progression in girls is faster than boys. More studies are needed in future to confirm this.

Our study revealed the high level of undercorrection of myopia, but this fails to account for amblyopia. Some studies showed that the prevalence of amblyopia in children is about 1.09-4.3 % [26–28], which can be used to roughly adjust the undercorrection of myopia. It’s noteworthy that non-cycloplegia refraction would still overestimate the myopia prevalence of children, the undercorrection rate of myopia will also be overestimated. But this data would provide the reference value for future studies.

The total SER remained stable from 3 to 6 years old, but it drifted towards myopia thereafter (+ 0.22 D and − 2.71 D at the ages of 6 and 14 years, respectively). The total SER in the girls was more myopic than that in the boys. The study showed a trend that the SER in the girls was more myopic than that in the boys aged 9 years and older. Thorn et al. [14] found that the SER of girls was more myopic than that of boys in grade 5 and grade 6 (mean difference of 0.26 D and 0.29 D in key schools and 0.16D and 0.10D in non-key schools, respectively). This result was slightly inconsistent with that in our study (mean difference of 0.15D and 0.22D at the ages of 10 and 11). This inconsistency may be because we did not distinguish the key and non-key schools. At the age of 13, the SER in the girls (-2.43 D) was more myopic than that in the boys (-2.30 D), but the difference was not significant. This may be due to the insufficient sample size. The difference in SER (0.37 D) between the girls and boys reached clinical significance at 14 years old.

In the study, AL increased with age (21.93 mm and 24.64 mm at the ages of 3 and 14, respectively), except between 4 and 5 years old. When the children developed myopia, the AL reached 23.54 mm (23.79 mm in the boys and 23.28 mm in the girls), whereas the AL is generally considered about 24 mm in emmetropic adults [29, 30]. The total AL in the boys was longer than that in the girls, which was consistent with the findings of previous studies [31–33]. Moreover, from the age of 3 to 14 years, the AL in the boys was always longer than that in the girls.

CR at the age of 3 was steeper than that at any other age and remained stable after the age of 4. It was also found that the CR in the girls was steeper than that in the boys, even from the age of 3. Scheiman [34] showed that girls had a steeper CR than did the boys, which was consistent with the findings of our study. He found that the corneal curvature slightly but significantly flattened over 14 years, but we did not observe this. This discrepancy might be due to differences in the study design and ethnicity of the study population.

Considering that the AL/CR ratio strongly correlated with SER, some studies have suggested that the AL/CR ratio could be an optimal indicator of myopia for detection [35–37]. AL/CR increased with age except between 4 and 5 years old. This finding was similar to the change in AL, implying that AL is the main morphological element related to myopia. Blanco proposed that corneal function possibly compensates for the slight increase in axial length due to myopization. When the axial length increased excessively, this effect of the cornea seemed to disappear [38]. This finding also indicates that myopia is mainly axial in children. The cut-off point of AL/CR with appearance of myopia was 3.00 at the age of 9 in our study, which was similar to that reported in previous studies [35, 36]. Moreover, AL/CR was higher in the boys than in the girls from the age of 4, except at the ages of 5 and 14. However, we found that the AL/CR ratio was higher in the boys than in the girls, even at the ages of 5 and 14, which may be due to the insufficient sample size. More studies are needed to confirm this hypothesis in the future.

## Limitations

It should be noted that there are some limitations of our study. Refraction was measured without cycloplegia, even though visual acuity was also measured, but the prevalence of myopia may have been overestimated. This study is a cross-sectional study rather than a longitudinal study, which reduces the value of the study to a certain extent. The small population of children aged 3–5 years may have an impact on some results in the study. However, presenting the data of children aged 3–5 years will provide some basis for future comparisons.

## Conclusions

There is a strikingly high prevalence of myopia in children aged 3 to 14 years old, but the fully corrected rate of myopia is very low, less than one-fifth of corrected myopic children. The prevalence of myopia in girls is higher than that in boys. However, AL and AL/CR in boys are higher than those in girls. With increasing age, the prevalence of myopia increases, and SER drifts to be more myopic. The high myopia rate in children is also rising, so it is necessary to take appropriate myopia prevention and control actions to retard the rapid progression of myopia.

## Data Availability

The datasets generated during and analysed during the current study are not available due to the protection of data security (the original data contains a lot of specifically demographic characteristics information and will be used again in the future follow-up study) but are available from the corresponding author on reasonable request.

## Abbreviations

VA: Visual acuity
SER: Spherical equivalent error
AL: Axial length
CR: Corneal radius
D: Dioptre
N: Number
mm: Millimeter
N1: The number of myopic children
N2: The total number of children
